# Static Magnetic Field Induced Neural Stem/Progenitor Cell Early Differentiation and Promotes Maturation

**DOI:** 10.1155/2019/8790176

**Published:** 2019-10-16

**Authors:** Shih-Yin Ho, I-Chun Chen, Yi-Jyun Chen, Chien-Hsing Lee, Chao-Ming Fu, Fei-Chih Liu, Horng-Huei Liou

**Affiliations:** ^1^Department of Neurology, National Taiwan University Hospital and National Taiwan University College of Medicine, Taipei 10051, Taiwan; ^2^Department and Graduate Institute of Pharmacology, College of Medicine, National Taiwan University, Taipei 10051, Taiwan; ^3^Department of Pharmacology, Graduate Institute of Medicine, College of Medicine, Kaohsiung Medical University, Kaohsiung 80708, Taiwan; ^4^Department of Medical Research, Kaohsiung Medical University Hospital, Kaohsiung 80708, Taiwan; ^5^Department of Physics, National Taiwan University, Taipei 10617, Taiwan; ^6^National Taiwan University Hospital Yunlin Branch, Douliu 64041, Taiwan

## Abstract

The potential impacts of magnetic field exposures on brain development have raised public concern. In the present study, we aimed to investigate the biophysical effects of moderate-intensity (0.5 T, Tesla) static magnetic field (SMF) on mice neural progenitor cells (mNPCs). Our results showed that the SMF exposure increased the number of neurosphere formation and enhanced proliferative activity in mNPCs. In addition, our flow cytometry data demonstrated that the proportions of S phase and G2/M phase mNPCs were remarkably increased following 5 days of SMF exposure. Moreover, the level of a mitotic regulatory protein, cyclin B, was upregulated after SMF exposure. Furthermore, the mNPCs exposed to SMF exhibited a significant increase in Sox2 expression. When mNPCs were induced to differentiation, our immunofluorescence assay revealed that the percentage of neurons (Tuj-1-positive cells) but not astrocyte (s100*β*-positive cells) was significantly higher and displayed morphological complexity in the SMF group. Finally, our electrophysiological results demonstrated the mNPC-derived neurons from the SMF group showing a significantly increased in input resistance, which indicated more functional maturation. Based on these findings, it appears reasonable to suggest that SMF exposure could affect normal neurogenesis and promote neural lineage differentiation as well as neuronal maturation.

## 1. Introduction

In recent years, there has been a dramatic increment of research concerned with the influence of the magnetic field (MF) on biological functions, especially on the central nervous system. For example, extremely low-frequency electromagnetic field (ELF-EMF), emitted from electronic devices, has been well reported to exert multiple modulating effects on the nervous system such as hippocampal neurogenesis, synaptic plasticity, and neuronal apoptosis [1–5].

MF can either be static or time-varying (electromagnetic). Static magnetic field (SMF), in particular, is more user-friendly to apply in clinical therapy, because only simple magnet is used to generate SMF. SMF is constant, time-independent, and zero-frequency MF, which does not change in direction or magnitude over time. According to its intensity, SMF can be classified as weak (<1  mT), moderate (1  mT–1  T), strong (1–5  T), and ultrastrong (>5  T) [6]. Although the therapeutic effect of different strength SMF in clinic has been demonstrated, however, there are contradictory studies that indicate that the SMF may increase the risks of health [7]. Therefore, it is important to explore the effects of the certain strength SMF on physiological functions.

Recently, the study by Prasad et al. [8] demonstrated that 2-week 0.3 T moderate SMF stimulation promotes differentiation of human oligodendrocytes precursor cells. In addition, the other study by Ammari et al. [9] indicated that mice exposed to a moderate-intensity (128 mT) SMF for 5 consecutive days caused learning impairment in the Morris water maze performance (a hippocampal-dependent spatial memory task). These results suggested that repeated moderate SMF exposure may affect normal neurogenesis. Indeed, previous studies demonstrated that the sustained 100 mT SMF exposure could suppress rodent NPC proliferation and facilitate differentiation into neurons [10].

Neurogenesis is a highly dynamic process that regulated by numerous cell-intrinsic transcription factors or extrinsic microenvironment in the mammalian nervous system. It has been known that the unequal distributions of ions on both sides of every cell membrane could drive electrical potential and thus created an intense electric field between the intra- and extracellular compartments. According to Faraday's law of electromagnetic induction, MF can interact with an electric circuit to produce an electromotive force. Therefore, it is reasonable to propose that an external signal such as SMF, which in turn causes an artifactual current, can interfere with neurogenesis. Indeed, endogenous electrical currents have been found to play an important role in biological functions, including neurogenesis [11]. Furthermore, previous studies demonstrated that electrical stimulation in differentiating embryonic stem cells is known to promote fate determination toward neuronal lineages [12]. On the other hand, an increasing number of recent studies on ELF-EMF and SMF have shown that the MF can exert positive and negative influences on mNPCs by affected intracellular signaling or gene expression such as Ca^2+^, *Mash1*, *Math1*, *Math3*, *NeuroD2*, *Hes1*, and *Hes5* [1, 10, 13]. However, the exact mechanisms of SMF which interact with the individual cell are still unclear.

The present study is aimed at determining whether continuous exposure to moderate-intensity SMF affected the proliferation and differentiation of mNPCs. Here, we demonstrated that SMF exposure induced enhancement of cell proliferation, neuronal differentiation, and neuronal maturation of mNPCs. These results provide further evidence of the effects of moderate SMF exposure on mNPC development.

## 2. Materials and Methods

### 2.1. Animals

Wild-type neonatal ICR mice were obtained from the Animal Center of National Taiwan University (Taipei, Taiwan). All of the animal experiments were performed in accordance with guidelines established by the Institutional Animal Care and Use Committee of the National Taiwan University College of Medicine.

### 2.2. Neurosphere Culture

Neurosphere cultures were prepared following the procedures described in our previous study [14]. Briefly, neonatal ICR mice (1-2 days old) were sacrificed and the brains were quickly removed into chilled HEPES solution and dissected by a surgical blade. The brains were then incubated with digestion buffer and neutralized with 10% heat-inactivated fetal bovine serum (Invitrogen). Dissociated cells were resuspended at a density of 2 × 10^5^ cells/ml in serum-free DMEM/F12 medium (Gibco-BRL) and supplemented with N2 complement in the presence of 10 *μ*g/ml epidermal growth factor (EGF) and 20 *μ*g/ml of basic fibroblast growth factor (bFGF) at 37°C with 5% CO_2_. The primary culture was maintained for 6 days to allow development of proliferative neurospheres.

### 2.3. SMF Exposure

**Figure 1:**
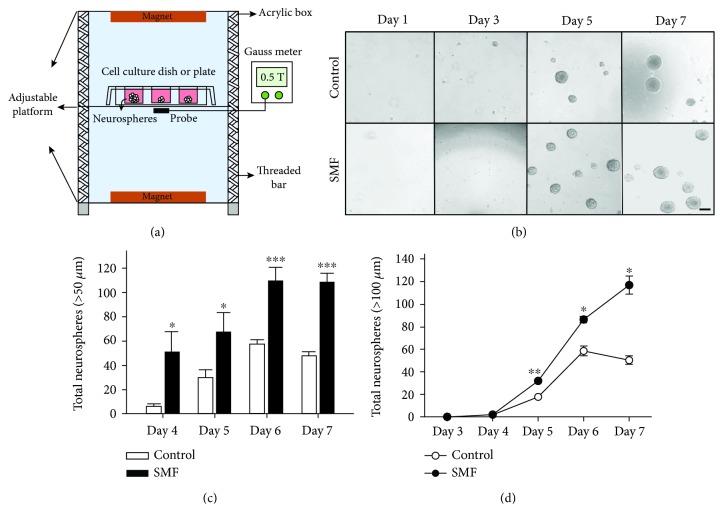
SMF stimulates the proliferation of mNPCs. Neurospheres developed from the neurosphere assay of control and SMF group. (a) Schematic drawing of the experimental device. (b) Typical phase-contrast micrographs. Scale bar, 100 *μ*m. (c) Neurosphere numbers per well developed from 4, 5, 6, and 7 day cultures. The number of neurospheres is significantly increased under SMF exposed. (d) Neurosphere numbers per well at sizes (>100 *μ*m) developed from 3-7 day cultures. Data represent the mean ± S.E.M.; control, *n* = 5; SMF, *n* = 3; ^∗^*p* < 0.05, ^∗∗^*p* < 0.01, ^∗∗∗^*p* < 0.001; Student's *t*-test.

The magnetic device was produced by a parallel pair of circular magnets positioned opposite each other, with the culture of mNPCs between them (Figure 1(a)). The field strength was measured with a Gauss meter (Lake Shore Model 420 Gauss meter, Lake Shore Cryotronics, Westerville, OH, USA) at the midpoint of the culture. The SMF induction values at the inner wall of the dish and flask were 0.51 ± 0.01 Tesla. The exposed and the control mNPCs were kept in the same incubator (37°C in 5% CO_2_ humidified atmosphere) but with at least 30 cm distance gap during the experiments. The magnetic induction values of the environmental geomagnetic field were also monitored and showed about 5 orders of magnitude lower than those inside cell containers.

### 2.4. Neurosphere Formation Assay

For the neurosphere formation assay, the neurospheres were dissociated into single mNPCs and reseeded in 96-well plate at a concentration of 2000 cells/well in 200 *μ*l of proliferation medium per well. The cells of the experimental group were exposed to SMF for 7 days (24 hours/day). Each day, both control and SMF exposure groups were taken out from the incubator for imagining. Whole well images were collected using an inverted microscope. All neurospheres contained in each well were captured and analyzed with ImageJ software (NIH).

### 2.5. Cell Cycle Analysis

To determine the distribution of mNPCs within G1, S, and G2/M phases of the cell cycle, a flow cytometry cell cycle analysis kit was used (Abcam) and the manufacturer's protocol was followed. Briefly, the neurospheres were dissociated into a single cell by accutase digestion and fixed in 75% ice-cold ethanol overnight at 4°C. The fixed cells were then stained with 50 *μ*g/ml propidium iodide (PI) containing RNase A for 30 min at room temperature. Cell fluorescence was assessed using FACSCAria II (BD Biosciences), and results were analyzed by FACSDiva software (BD Biosciences).

### 2.6. Western Blotting

Whole-cell extracts were isolated using a lysis buffer (20 mM Tris-HCl pH 8.0, 400 mM NaCl, 5 mM EDTA, 1 mM EDTA, 1 mM Na pyrophosphate, 1% Triton X-100, 10% glycerol). Proteins were fractionated by SDS-PAGE (12% gel) and transferred onto PVDF membranes (Amersham). After incubation with nonfat milk in Tris-buffered saline with Tween 20 (TBST) for 60 min, the membrane was washed once with TBST. Primary antibodies used in immunoblot experiments were as follows: goat anti-*α*-tubulin (1 : 1000, Abcam), mouse anti-Sox2 (1 : 1000, Millipore), mouse anti-Cyclin D (1 : 1000), mouse anti-Cyclin B, (1 : 1000), mouse anti-PCNA, (1 : 1000), and mouse anti-p21 (1 : 500) were all purchased from Cell Signaling Technology. Secondary antibodies (anti-goat, anti-mouse) conjugated with horseradish peroxidase were used (Santa Cruz Biotechnology). Reactions were developed by the enhanced chemiluminescence (ECL) procedure using luminol and coumaric acid as substrates and exposed on a Hyperfilm ECL (Amersham). Immunoblot images were quantitated using ImageJ software (NIH).

### 2.7. Immunocytochemistry

The neurospheres obtained from the control and SMF-treated groups were dissociated and reseeded in mitogen-free medium allowed to differentiate. After 7 days of differentiation, cells were washed with phosphate-buffered saline (PBS), followed by fixation with 4% paraformaldehyde for 20 min at 4°C. Then, the cells were reacted with primary antibodies adequately diluted against the neuronal marker Tuj-1 (1 : 100) and astrocytes marker GFAP (1 : 500) overnight at 4°C. After washing with PBS, appropriate secondary antibodies conjugated with Alexa Fluor 488 or 555 (1 : 500, Invitrogen) were applied for 1 hour at RT. Images were taken with a confocal laser scanning microscope (LSM780, Carl Zeiss).

### 2.8. Electrophysiology

For patch-clamp experiments, the neurospheres obtained from the control or SMF exposure groups were reseeded into fibronectin-coated coverslips in mitogen-free medium allowed to differentiate. After 3 weeks of differentiation, the coverslips were transferred into a bath chamber and constantly (2~3 ml/min) perfused with fresh recording medium containing (in mM): 2 CaCl_2_, 10 glucose, 3 KCl, 1 MgCl_2_, 125 NaCl, 26 NaHCO_3_, and 1.25 NaH_2_PO_4_. The whole-cell recording was made at room temperature (22°C), by using an Axopatch 200B amplifier and 1440A interface (Molecular Devices). Signals were filtered at 2 kHz using amplifier circuitry, sampled at 10 kHz, and analyzed using Clampex 10.2 (Molecular Devices). Patch electrodes (3-8 M*Ω*) were pulled from borosilicate glass capillaries by a micropipette puller (P97, Sutter Instrument) and filled with a solution containing (in mM): 140 K-Glu, 5 KCl, 10 HEPES, 0.2 EGTA, 2 MgCl_2_, 4 MgATP, 0.3 Na_2_GTP, and 10 Na_2_-phosphocreatine; pH 7.2 (with KOH). Action potential firing was recorded under current-clamp conditions. Membrane capacitance (Cap) was measured by calculating the total charge mobilized by 10 mV depolarizing steps.

### 2.9. Statistical Analysis

All experiments were performed at least three times with similar results. Data were shown as mean ± SEM and analyzed using SigmaPlot 10.0 software (Systat Software). The data were evaluated statistically by Student's *t*-test. Statistical significance was set at *p* < 0.05.

## 3. Results

### 3.1. SMF Exposure Enhances the Self-Renewal Ability of mNPCs

To determine whether the mNPC proliferation was affected by moderate-intensity SMF exposure, we first examined the cell proliferative capacity of mNPCs by neurosphere assay. As shown in Figure 1(b), the neurospheres obtained from neonatal mice were able to grow and become visible after a culture time of 3 days. These neurospheres were allowed to expand for 7 days *in vitro*. Interestingly, we found that both the number and size of floating neurospheres in the SMF group seem more evident than the untreated control group. We, therefore, quantified the number of different neurosphere sizes at day 3, 5, 6, and 7 culture times. Our results showed that there were significant increases in total neurosphere numbers after SMF stimulation (control/SMF: day 4, 6.02 ± 2.32/51.15 ± 16.89; day 5, 29.93 ± 6.20/67.44 ± 16.06; day 6, 57.46 ± 3.88/109.89 ± 10.83; day 7, 47.78 ± 3.38/108.47 ± 7.10; *p* < 0.05, *p* < 0.01, *p* < 0.001, Figure 1(c)). The neurosphere diameter has been known as an indicator of proliferative potential [15]. For this reason, we measured the growth curve of larger neurosphere population (>100 *μ*m) at a different time point after seeding. Our results showed that exposure to SMF induced a significant increase in larger neurosphere numbers at days 5, 6, and 7 after plating (control/SMF: day 5, 18.06 ± 2.52/31.89 ± 1.94; day 6, 58.53 ± 4.42/86.44 ± 2.62; day 7, 50.33 ± 3.68/117.11 ± 8.04; *p* < 0.05, *p* < 0.01, Figure 1(d)). Noticeably, we observed the outgrowth of neurospheres in the control group reached a plateau at day 6. However, the growth of large neurosphere population in the SMF group seems not to have reached a plateau stage on day 7 of culture.

### 3.2. Effects of SMF Exposure on the Cell Cycle and Cell Cycle-Related Proteins of mNPCs

**Figure 2:**
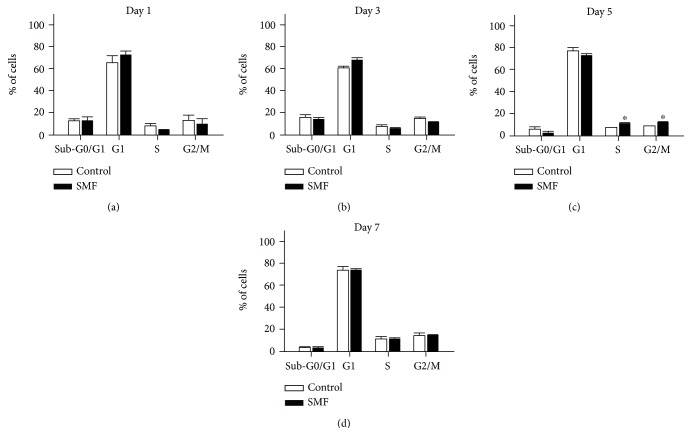
SMF increases self-renewal of mNPCs. Quantification of cell cycle profiles by FACS analysis in mNPC populations under SMF. SMF increased the percentage of cells in S and G2/M phase on day 5 *in vitro*. Data represent the mean ± S.E.M.; *n* = 2‐4 in each group; ^∗^*p* < 0.05; Student's *t*-test.

The cell cycle is a series of events involving cell growth and cell division. To examine whether the higher level proliferative cells may be triggered by the enhancement of cell cycle progression, we used the flow cytometry cell cycle analysis to calculate the cell cycle distribution after exposure to SMF. Our results revealed that the cell cycle distribution at 1, 3, and 7 days after SMF stimulation showed no significant changes (Figures 2(a), 2(b), and 2(d)). Interestingly, our quantitative assessment revealed a significantly increased percentage of cells in the S phase (control/SMF, 7.47 ± 0.02%/11.76 ± 0.48%; *p* < 0.05) and G2/M phase (control/SMF, 9.24 ± 0.34%/12.71 ± 0.43%; *p* < 0.05) at 5 days following SMF exposure (Figure 2(c)). Additionally, the PI flow cytometric analysis has been widely used for the assessment of apoptosis in many experimental models [16]. We, therefore, evaluated whether the mNPC viability was affected by SMF. As shown in Figure 2, our data showed the SMF exposure did not have apparent effects on the percentages of cells in Sub-G0/G1 phase between the control and SMF groups. These results demonstrated that the cell cycle progress of mNPCs was affected under SMF exposure.

**Figure 3:**
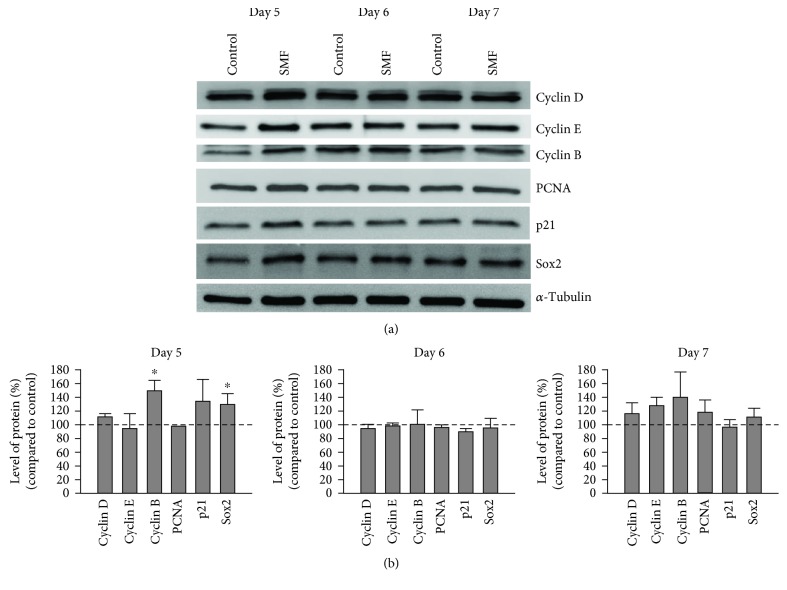
SMF mediated cell cycle progression of mNPCs. (a) Western blotting showing the level of cell cycle-related proteins expressed in mNPCs under SMF on days 5, 6, and 7. (b) Data in the graph represent fold changes, calculated relative to the values in the control groups, after 5, 6, and 7 days of SMF exposure. The levels of both cyclin B and Sox-2 in mNPCs were markedly increased after 5 day SMF. Blotting bands were analyzed using the ImageJ software for relative density and normalized to *α*-tubulin controls. Data represent the mean ± S.E.M.; *n* = 2‐5 in each group; ^∗^*p* < 0.05; Student's *t*-test.

To further explore the molecular mechanism of mNPC cell cycle regulation under SMF exposure, we then harvested the mNPCs after SMF exposure and subjected to western blot analysis. We detect the protein expression of cell cycle-related proteins, including cyclins (cyclin B, D, and E), PCNA (proliferating cell nuclear antigen), and p21. As shown in Figures 3(a) and 3(b), we found the cyclin B was significantly increased after SMF exposure for 5 days (149.12 ± 15.39% compared to control; *p* < 0.05). Overall, our results showed that the exposure to SMF increase proliferation of mNPCs is regulated by the cell cycle through mechanisms involving cyclin B.

### 3.3. Effects of SMF Exposure on the Differentiation of mNPCs

**Figure 4:**
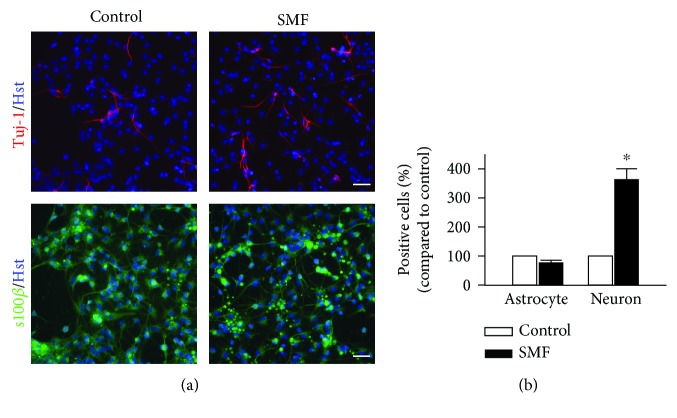
SMF exposure promotes fate determination toward neuronal lineages and neuronal maturation of mNPCs. (a) The mNPC-derived cells were stained with an antibody for Tuj-1 (red), s100*β* (green), and Hoechst 33342 (Hst, blue). The immunostaining images showed that the percentage of Tuj-1-positive cells was significantly increased after SMF stimulation. Scale bar, 25 *μ*m. (b) Summary of the change of s100*β*- and Tuj-1-positive cells in cultures that treated/untreated with SMF. ^∗∗^*p* < 0.05 compared with control groups (Student's unpaired *t*-test).

MF has been reported to enhance the neuronal differentiation of cortical mNPCs *in vitro* [17]. To evaluate whether SMF affected the cell fate determination of mNPCs, we used the immunocytochemical detection to analyze the neuronal or astroglial cell populations derived from differentiating mNPC progeny. Our results demonstrated that the percentage of Tuj-1 (neuronal marker)-positive cells in the SMF group was significantly higher than the untreated control group. In contrast, our analysis showed no difference in the s100*β* (astroglial marker)-positive cells between the SMF and control groups (75.44 ± 8.77% and 361.54 ± 38.46% for astrocyte and neuron population, respectively; Student's unpaired *t*-test; *p* < 0.05, Figures 4(a) and 4(b)). These findings indicated that mNPCs cultured after exposure to SMF were more likely to give birth to neurons. It is worth noting that the expression levels of Sox-2 were also significantly higher with SMF exposure (129.33 ± 15.72% compared to control; *p* < 0.05, Figures 3(a) and 3(b)). Recently, it has been suggested that the transcription factors Sox-2 not only play a critical role in the maintenance of pluripotency but also in functioning as the neuronal lineage-specific regulator [18]. Taken together, our data indicated that SMF exposure can promote mNPC differentiation toward neuronal lineage.

### 3.4. Effects of SMF Exposure on the Morphology and Electrophysiology of mNPC-Derived Neurons

**Figure 5:**
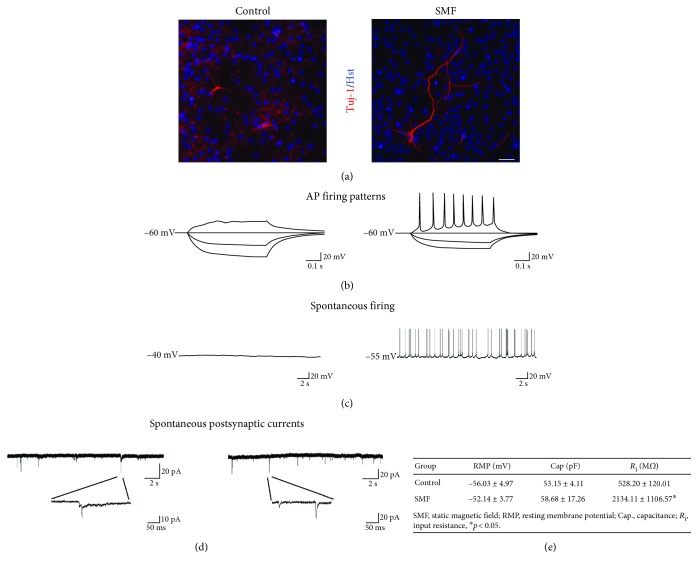
Electrophysiological characterization of the mNPC-derived neuron. (a) Representative images showing the Tuj1 staining for the neurite outgrowth of mNPC-derived neurons. SMF stimulation (right panel) markedly increased the length and the branching of neurites than control neuron (left panel). Scale bar, 25 *μ*m. (b) Responses of the mNPC-derived neuron to current injections (-60, -30, 0, +30 pA, duration, 500 ms, holding potential, -60 mV). (c) Representative current-clamp recordings of the mNPC-derived neuron. After exposure to SMF, mNPC-derived neuron exhibited spontaneous repetitive AP firing. (d) Representative examples of spontaneous postsynaptic currents at 3 weeks after differentiation (recordings were taken without blockers of synaptic transmission). Both control and SMF groups are showing spontaneous postsynaptic currents. (e) mNPC-derived neuron displayed a significantly increased in *R*_i_ but not RMP or Cap after exposure to SMF. ^∗^*p* < 0.05; Student's *t*-test.

To test whether the mNPC-derived neurons were influenced by SMF exposure, we performed the morphological analysis and whole-cell patch recordings in order to examine their maturation and electrophysiological characteristics. The results from our immunocytochemical staining showed that the mNPC-derived neurons from the SMF group exhibited higher neurite complexity than the control group (Figure 5(a)), suggesting SMF exposure promotes the neurite extension and neuronal morphological maturation of mNPC-derived neurons. Additionally, after 3 weeks of postdifferentiation, we found the action potential (AP) firing was only observed in the SMF groups following superthreshold current injection. Moreover, these mNPC-derived neurons exhibited more mature spontaneous repetitive AP firing (Figures 5(b) and 5(c)). We next investigated the basic synaptic transmission in mNPC-derived neurons. Both control and SMF groups shown spontaneous postsynaptic activity. However, we found that there were not significant differences between the control and SMF groups on spontaneous postsynaptic currents (Figure 5(d)). Furthermore, we also assessed the passive membrane properties of mNPC-derived neurons following MF exposure. As shown in Figure 5(e), mNPCs displayed a significantly increased in input resistance (*R*_i_) but not resting membrane potential (RMP) or capacitance (Cap) after exposure to SMF (control, RMP −56.03 ± 4.97(*n* = 14), Cap 53.15 ± 4.11 (*n* = 8), *R*_i_528.20 ± 120.01 (*n* = 8); SMF, RMP −52.14 ± 3.77 (*n* = 17), Cap 58.68 ± 17.26 (*n* = 3), *R*_i_2134.11 ± 1106.57 (*n* = 3), *p* < 0.05). These data suggest that SMF exposure during the mNPC proliferative period may affect subsequent differentiation and promote neuronal maturation.

## 4. Discussion

**Figure 6:**
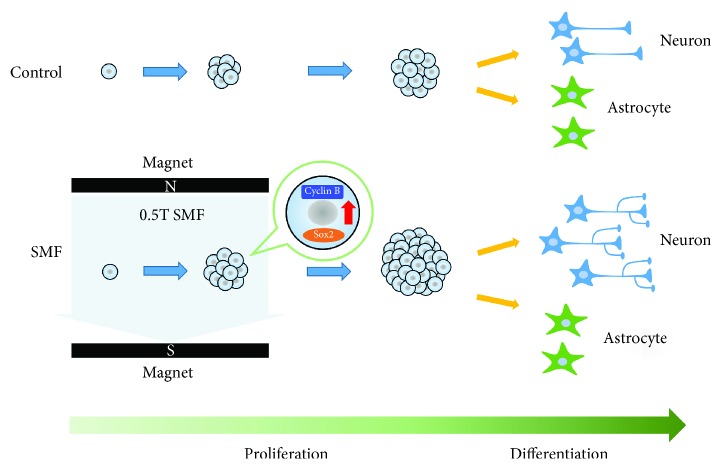
Schematic representation. SMF exposure stimulates mNPC proliferation through upregulation of cyclin B and Sox-2 required for self-replication. Moreover, SMF also promotes mNPC fate determination toward neuronal lineages and neuronal maturation.

Our study showed that exposure to moderate-intensity SMF enhances the proliferation potential of mNPCs. The S and G2/M phase of mNPCs increased under the SMF exposure compared with the control groups. In addition, cells that had higher levels of Sox2 and cyclin B expressions follow SMF stimulation. Furthermore, SMF exposure induced mNPC preferred differentiation into neurons and displayed a significant increase in degrees of morphological and electrophysiological maturity (Figure 6). Our results supported that SMF exposure may not only affect mNPC proliferation (associated with cell cycle progression) but also influence its fate determination and subsequent maturation.

It is now clear that MF can induce biological changes in mNPCs (for review on MF and mNPCs, see [19–21]). The extensive *in vivo* and *in vitro* studies suggested that the MF exposure can enhance mNPC proliferation. For example, adult mice received ELF-EMF stimulation for 7 consecutive days (1-7 h/day) causes a significant increase in the number of BrdU^+^/DCX^+^ cells in the hippocampal dentate gyrus [1]. In addition, exposed to an ELF-EMF on *in vitro* cultured mNPCs at different time intervals also results in a remarkable enhancement in cell proliferation [22, 23]. These results indicated that the MF exposure could potentially induce a physiological influence on neurogenesis. In the present study, we observed that mNPCs from the SMF groups generated significantly larger neurospheres compared with the control groups (Figures 1(b) and 1(c)). Besides, the proliferation rate of large neurospheres was more rapidly under SMF stimulation (Figure 1(d)). Our results, therefore, suggested that SMF exposure accelerated proliferative activity in mNPCs. The mechanisms of the MF on mNPC proliferation have been reported in many studies. For instance, Leone et al. [24] demonstrated that the ELF-EMF-induced proliferation of mNPCs is associated with the upregulation of the Hes1 (pro-proliferative gene) expression, which is caused by the enhancement of Ca_v_1 channel-dependent H3K9 acetylation on Hes1 promoter region. Furthermore, Cheng et al. [22] reported that the Akt signaling pathway is involved in ELF-EMF-induced proliferation in ischemic mNPCs. Even though the present work did not explore directly how SMF exposure affects mNPC proliferation, our results clearly showed the SMF exposure could change mNPC cell cycle progression. The size of neurospheres has been known as an indicator of proliferative potential [15], which is generally assumed to be correlated with the cell cycle. Generally, the cell cycle of murine neurospheres slowly and rarely undergoes mitosis at any time. Previous studies revealed that large parts of the mNPCs have stayed in the G1 phase [17, 25]. In agreement with these findings, our flow cytometry results showed that approximately 60–80% of mNPCs were detected in the G1 stage (Figure 2). Although we did not find any significant difference in the cell cycle distribution of mNPCs after 1, 3, and 7 days of SMF exposures, we observed a remarkable increase of mNPCs in S phase and G2/M phase following 5 days of SMF stimulation. Therefore, there might be a critical time window for the mNPC proliferations under SMF exposure. Our data are partially consistent with previous work on mNPCs, which showed no significant change in cell cycle distribution after 3 days of ELF-EMF exposure [17]. Interestingly, we also observed a G2/M phase corresponding cell cycle-associated protein-cyclin B increase in the mNPCs at 5 days of SMF treatment. These results implied that the MF may have the potential to modulate a cell cycle regulator. Indeed, the experiment from a rat ischemia model demonstrated that 10 Hz rTMS (repetitive transcranial magnetic stimulation) stimulation can promote the proliferation of adult NPCs by regulating the miR-25/p57 signaling cascade [26].

In the current study, we also found highly elevated levels of Sox2 in the mNPCs at 5 days but not 6 days and 7 days of the SMF exposure groups (Figure 3(b)). The Sox2 transcription factor has been reported to be expressed at high levels in mNPCs in both embryonic and adult brains [27]. Deletion of Sox2 in mNPCs led to a decrease in neural progenitor populations suggesting that Sox2 is important for maintaining mNPCs in the developing brain. Accordingly, this phenomenon is due to a change in the neurosphere characteristics. Neurospheres are heterogeneous structure, and this heterogeneity increases with its sphere size. When the neurospheres grow bigger under continuous SMF exposure, the mNPCs in the core of neurospheres are unable to acquire sufficient mitogens and initiate differentiation. On the other hand, although the functions of Sox2 in the maintenance of stem cells in an undifferentiated state are well defined, other studies proved that Sox2 is also required for the formation and maturation of differentiated neurons [18]. For example, conditional loss of function of Sox2 in mNPCs did not affect the self-renewal ability of mNPCs but reduced its neurogenic capacity [28]. Moreover, the mNPCs obtained from Sox2-deficient mice demonstrated that the proliferative potential of mNPCs was less unaffected but severely impaired to neuronal differentiation (most of the neurons failed to develop into maturity and showed morphological abnormalities) [29]. In contrast, several studies have shown that overexpression of Sox2 could reprogram astrocytes into the neuron. For instance, forced expression of Sox2 alone was sufficient to induce the conversion of NG2 glial cells into neurons in the adult mouse cerebral cortex following injury *in vivo* [30]. Interestingly, Cimadamore et al. [31] demonstrated that SOX2 is required for human embryonic stem cell-derived NPC neuronal differentiation. They found that SOX2 drives the expression of the proneuronal genes *MASH1* and *NGN1* by directly binding the promoter region of these genes. The high levels of Sox2 may, therefore, repress mNPCs toward astroglia cell fate and promote neurogenic differentiation. Our results demonstrated that the percentage of neurons (but not astrocytes) was significantly higher in the SMF group while exposed to SMF for 7 days (Figure 4(b)). These findings are consistent with the results of previous reports stating that MF exposure such as SMF and ELF-EMF could promote murine NPC differentiation toward neuronal lineage through the upregulation of proneuronal genes (*Mash1*, *Math1*, *Math3*, *Ngn1*, and *NeuroD*) [1, 10, 23, 24]. However, further studies are required to determine the relationship between Sox2 and proneuronal genes under SMF exposures.

Additionally, our immunocytochemical staining data demonstrated that the mNPC-derived neurons exhibited a tendency to develop more maturity after SMF exposure (Figure 5(a)). These results are paralleled with the previous study that ELF-EMF exposure promotes the neurite outgrowth in mNPC-derived neurons by increasing the TRPC1 and proneuronal genes expression [23]. In association with morphological maturation, we found the mNPC-derived neurons from the SMF group also exhibited mature repetitive AP firing upon superthreshold current injection (Figure 5(b)). This finding suggested that the neurons from the SMF group are more functional maturity as previously reported in human pluripotent stem cell-derived neurons [32]. The spontaneous AP firing is an important characteristic of neuronal network maturity [33]. Similarly, we found that the mNPC-derived neurons develop a spontaneous repetitive AP firing in the SMF group but not the control group (Figure 5(c)). Another important criterion of neuronal maturation is synaptic connectivity. Interestingly, we observed the spontaneous postsynaptic activity was evident in both control and SMF groups (Figure 5(d)). A possible explanation for this result might be the spontaneous neurotransmitter release early in neuronal development [34]. Along with more mature firing properties, we also found a significantly higher *R*_i_ in the mNPC-derived neurons under SMF exposure (Figure 5(e)). The relatively higher *R*_i_ represents the more functional maturity which may cause by electrical uncoupling to other cells as previously described in cortical development [35, 36]. Taken together, our results reveal that Sox2 may be a key regulator to mediate proliferation and differentiation of mNPCs after exposure to SMF.

## 5. Conclusion

In conclusion, our study found that the 0.5 T SMF exposure could affect the biological properties and functions of mNPCs. Our results provide the novel evidence for the role of moderate-intensity SMF in mNPC development. Besides, our data offer an effective strategy for enhancing the neurogenic potential of mNPCs *in vitro*, which might help for the development of therapeutic approaches in neuroregenerative medicine in the future.

## Data Availability

The data used to support the findings of this study are available from the corresponding author upon request.
